# Hidden and Delayed-Onset Hearing Loss in Children: Limitations of Newborn Hearing Screening and Future Directions

**DOI:** 10.3390/children13091179

**Published:** 2026-09-01

**Authors:** Alenka Kravos

**Affiliations:** 1 Department of Otorhinolaryngology, University Medical Centre Maribor, Ljubljanska Ulica 5, 2000 Maribor, Slovenia; kravosalenka@gmail.com; Tel.: +386-41-596-484; 2 Faculty of Medicine, University of Maribor, Taborska Ulica 8, 2000 Maribor, Slovenia

**Keywords:** hearing loss, universal newborn hearing screening, diagnostic failure, etiology, auditory neuropathy spectrum disorders, congenital cytomegalovirus infection, genetic sensorineural hearing loss, auditory brainstem response, otoacoustic emissions

## Abstract

**Highlights:**

**What are the main findings?**

- Current universal newborn hearing screening (UNHS) fails to detect a substantial proportion of hidden, mild, progressive, delayed-onset, and neural hearing loss, even when neonatal screening results are normal.
- False-negative screening results are mainly associated with auditory neuropathy spectrum disorder (ANSD), genetic hearing loss (e.g., GJB2 and SLC26A4 mutations), congenital cytomegalovirus infection (cCMV), and auditory pathway immaturity, whereas false-positive results are largely caused by transient conductive conditions.

**What are the implications of the main findings?**

- Newborn hearing screening protocols should evolve beyond conventional OAE-based approaches by incorporating broader use of AABR, optimized screening timing, and long-term audiological follow-up to improve diagnostic accuracy.
- Integration of electrophysiological testing with genetic and congenital CMV screening may enable earlier identification of children at risk for hidden or delayed-onset hearing loss, thereby improving language, cognitive, and developmental outcomes.

**Abstract:**

**Background/Objectives**: Universal newborn hearing screening (UNHS) has significantly improved early detection of congenital hearing loss; however, discrepancies persist between prevalence at birth and latter childhood. These findings suggest the presence of hidden or delayed-onset forms of hearing impairments as a consequence of false-negative UNHS results. **Methods**: A bibliographic search was conducted in PubMed and Scopus for the period 2000–2026. A total of 103 papers met the inclusion criteria. This narrative review synthesizes recent literature on the limitations of current hearing screening strategies, focusing on false-positive and false-negative screening results, auditory neuropathy spectrum disorder (ANSD), genetic etiologies, and congenital cytomegalovirus (cCMV) infection as potential causes. Emphasis is placed on mechanisms underlying missed diagnoses and their clinical implications. **Results**: Current screening protocols, particularly those based on otoacoustic emissions (OAE), may fail to detect mild, progressive, or neural hearing loss. False-negative results are frequently associated with genetic mutations (e.g., *GJB2*), cCMV infection, and ANSD, whereas false-positive findings are often associated with transient middle ear conditions and early timing of testing. Automated auditory brainstem responses (AABR)-based approaches for hearing screening improve detection of neural dysfunction but are influenced by auditory pathway maturation, which may complicate interpretation of results in early life. Evidence indicates that a substantial proportion of sensorineural hearing loss identified later in childhood may not be detectable at birth using existing protocols. **Conclusions**: Hidden and delayed-onset sensorineural hearing loss represent significant challenges and limitations of current UNHS programs. Optimization of screening strategies—including broader implementation of AABR, integration of genetic and cCMV testing, and careful consideration of screening timing—may improve early detection of hearing loss and reduce long-term developmental consequences. Future updates of international guidelines should address these gaps to enhance diagnostic accuracy and clinical outcomes.

## 1. Introduction

Hearing is vital for the development of speech, language, cognitive abilities, and social skills in children [1,2,3]. Early detection of hearing loss is a fundamental public health task [4]. Before the introduction of universal newborn hearing screening (UNHS), sensorineural hearing loss was often not recognized until significant developmental delays became apparent [5], while milder forms frequently remained undetected even into adulthood [6].

Hearing loss is the most common congenital disorder, with an incidence of approximately 1 per 1000 newborns for severe hearing loss and deafness, and 4 per 1000 for mild to moderate hearing loss [7,8]. The Joint Committee on Infant Hearing (JCIH), established in 1969, has continually updated guidelines for the early detection of hearing loss and promoted adherence to the 1-3-6 principles (screening by 1 month, diagnosis by 3 months, and intervention by 6 months). These efforts form the foundation of modern early detection and intervention frameworks and have significantly improved early diagnosis [8,9]. The Committee was established at the initiative of the American Academy of Pediatrics, the American Academy of Ophthalmology and Otolaryngology, and the American Speech-Language-Hearing Association, which were among the first to recognize the importance of newborn hearing screening and continue to monitor, analyze, and guide the development of early detection of hearing loss [10]. Based on their initial studies, they found that the highest incidence of hearing loss was among children born with risk factors for hearing loss, and they initially conducted screenings only in this group [11]. The range of risk factors has continued to expand with the development of new diagnostic methods and, above all, with advances in medicine that have increased survival among even the most vulnerable newborns. Because many infants still had permanent hearing loss despite the absence of risk factors, UNHS was introduced in 1993 to include all newborns, thereby laying the foundation for today’s screening successes [12].

### 1.1. Discrepancy in the Incidence of Hearing Loss Immediately After Birth and During School Years

Despite the introduction of UNHS, a discrepancy remains between the prevalence of hearing loss at birth and later in childhood [13,14]. Some children with initially normal screening results later present with difficulties in sound perception, speech development, and communication, suggesting hidden or gradual hearing loss. The prevalence of permanent hearing loss gradually increases from birth through the school-age period. Although permanent hearing loss occurs in 1–2 per 1000 newborns, the prevalence rises to 3–6 per 1000 school-age children when delayed-onset, progressive, mild, and unilateral hearing loss are also included [15,16].

### 1.2. Hidden Reasons for Hearing Loss

Recently, new findings have emerged that deepen our understanding of these differences. These include auditory neuropathy spectrum disorder (ANSD) [17], progressive and delayed-onset hearing loss [18], genetic factors (including penetrance and epigenetic influences) [19,20], and congenital cytomegalovirus (cCMV) infection, which can cause subsequent clinically perceptible hearing loss [21]. Furthermore, maturation of the auditory pathway in newborns plays an important role, as it can affect screening test results and their reliability [22,23]. These findings have led to the concept of “hidden hearing loss,” which can significantly affect a child’s development despite initially normal screening results. They also raise questions about the adequacy of current screening strategies, including the timing of testing and the choice of diagnostic methods [24].

Although universal newborn hearing screening has substantially improved the early detection of congenital hearing loss, increasing evidence indicates that a significant proportion of children with hearing impairment are still not identified during the neonatal period. Unlike previous reviews that primarily describe newborn hearing screening techniques or individual etiologies of childhood hearing loss, this review proposes an integrated conceptual framework explaining why hidden and delayed-onset hearing loss remains undetected despite successful implementation of universal newborn hearing screening. This review aims to address this important knowledge gap by providing an integrated overview of the mechanisms responsible for false-negative and false-positive screening results and by discussing future directions that may improve early detection of hearing loss and long-term developmental outcomes.

## 2. Methods

This manuscript was conceived as a narrative review and therefore did not follow the PRISMA guidelines for systematic reviews. Nevertheless, a structured and transparent literature search and selection process was undertaken to identify the most relevant evidence on hidden and delayed-onset hearing loss in children. A bibliographic search was conducted in PubMed and Scopus for the period 2000–2026. The search strategy combined the following keywords: hearing loss; universal newborn hearing screening; diagnostic failure; etiology; auditory neuropathy spectrum disorders; congenital cytomegalovirus infection; genetic sensorineural hearing loss; auditory brainstem response; and otoacoustic emissions. A total of 235 papers written in English were identified. The inclusion criteria were original peer-reviewed research studies on the diagnostics and consequences of hearing loss, or review papers on the etiology and epidemiology of hearing loss, published in reputable professional or scientific journals, not supported or funded by parties who could influence the study results, and written by respected experts from the field. Relevant position papers with guidelines were also included. After removing ineligible papers, duplicates, and papers that did not meet the inclusion criteria or were not relevant to the topic, 103 papers remained.

The results of this narrative literature review are presented according to key topics.

## 3. Epidemiology of Hearing Loss

Hearing loss in children is a significant global problem. In newborns, the incidence of severe hearing loss and total deafness is approximately 1 in 1000. As children grow, the prevalence increases as additional hearing impairments are identified. These are not limited to impairments resulting from ear infections, meningitis, otitis media with effusion, congenital anatomical anomalies, or childhood head injuries—which primarily cause moderate hearing loss—but most newly identified impairments are mild and cannot be explained by these conditions. Prevalence rates for hearing loss vary widely [13], even within the same age group, with considerable differences across studies during adolescence. One study reports a prevalence of 14% [25], another 13.1% [26], and a third 20% [8]. In any case, the prevalence is higher than that observed in newborns.

### 3.1. Causes of the Rising Prevalence of Hearing Loss in Children and Adolescents

The following factors play a significant role in this increase:

- Advances in neonatology have led to higher survival rates among premature infants and children with severe congenital diseases, which in turn increases the risk of hearing loss. This has resulted in a larger population of children with known RF for hearing impairment. In cases of low birth weight, the prevalence of hearing impairment is 3.9% [27]; similarly, the prevalence of hearing loss is further increased by stays in intensive care units, where premature infants spend more time after birth, and reaches 2–3% [28].
- Childhood infections and diseases, including complications at birth, postnatal infections, ototoxic medications used to treat infections, otitis, meningitis, and CMV infection, the incidence of which is not necessarily increasing. However, modern treatment methods have led to more surviving children who also experience hearing-related consequences [8].
- Genetic and congenital causes of hearing loss are more common due to lower perinatal mortality among children with risk factors for hearing loss [20].
- Environmental factors, such as noise—especially among adolescents who are regularly exposed to noise from headphones when listening to music. Earphones used for listening to music are becoming increasingly popular [29].
- Improved diagnostics and screening programs for hearing loss can now detect what previously could not be detected [8].

However, the impact of progressive genetic, infectious and other factors, including ANSD, has often been overlooked in discussing the increased prevalence of hearing loss. Clinical manifestations of hearing loss caused by these factors can appear at very different ages in children, leading to confusion in the statistics and, consequently, highly variable data. For school-age children, reported rates range from 0.2% to 7.8% [8], but some authors find them to be as much as 50–90% higher at age 9 than at birth [30].

### 3.2. Hearing Loss Is Becoming a Global Problem in Children and Adults

In developed countries, UNHS is a standard part of newborn care, whereas in developing countries, hearing impairments are often not detected until age 5 or 6, by which point the developmental consequences are already irreversible. The World Health Organization (WHO) report analyzes in detail how UNHS covers nearly 100% of newborns in developed countries, whereas in developing regions this rate often falls below 10%, leading to vast disparities in early diagnosis and, consequently, quality of life [31]. In developing countries, a significantly higher proportion of hearing impairments is caused by treatable diseases (e.g., chronic middle ear infections and measles), which have been nearly eradicated or are successfully treated in the developed world. Access to cochlear implants and modern hearing aids in less and moderately developed countries is limited to only the wealthiest segment of the population.

As mentioned, the prevalence of hearing loss is increasing among schoolchildren and youth. It was reported that among Americans aged 12 and older, the prevalence is reaching up to 23% of the total population [32]. Children with hearing loss—including those with undiagnosed hearing loss—experience not only cognitive deficits but also reduced academic performance [24]. For this reason, it is even more important to identify all types of hearing loss through universal screening and to provide appropriate rehabilitation as soon as possible.

It is not only children who suffer the consequences of hearing loss; adults do as well. In fact, one-third of adults over the age of 50 experience mental health issues due to hearing loss [33]. Sheffield and Smith point out that the majority (approximately 80%) of all people with hearing loss live in low- and middle-income countries, where there is a shortage of audiological equipment, trained professionals, and financial resources for hearing rehabilitation (e.g., hearing aids) [20].

It is unclear whether the increasing number of adults with hearing loss is also due to the rising number of children with hearing loss. According to the WHO, there are already 32 million children with hearing loss worldwide [31]. By 2019, it was estimated that one in five people globally had a hearing impairment [34], with 62.1% of those over the age of 50 [32]. While most of the world’s population with hearing impairment has mild impairment [35], many people over the age of 80 are estimated to have moderate hearing loss [32].

For this reason, the WHO has declared hearing loss to be the third most common human condition and, at the same time, the most common sensory deficit [31]. At least 5% of the world’s population is estimated to require hearing rehabilitation [31]. WHO, therefore, recommends that countries increase the availability of hearing rehabilitation services by 20% by 2030 [31]. They also call on less developed countries to establish screening programs for children and adults.

The existing gap between developed and developing countries (low- and middle-income countries) is a central theme in WHO reports, as access to hearing screening programs and rehabilitation services is often severely limited in less-developed settings.

## 4. Development and Global Rollout of UNHS

**Figure 1:**
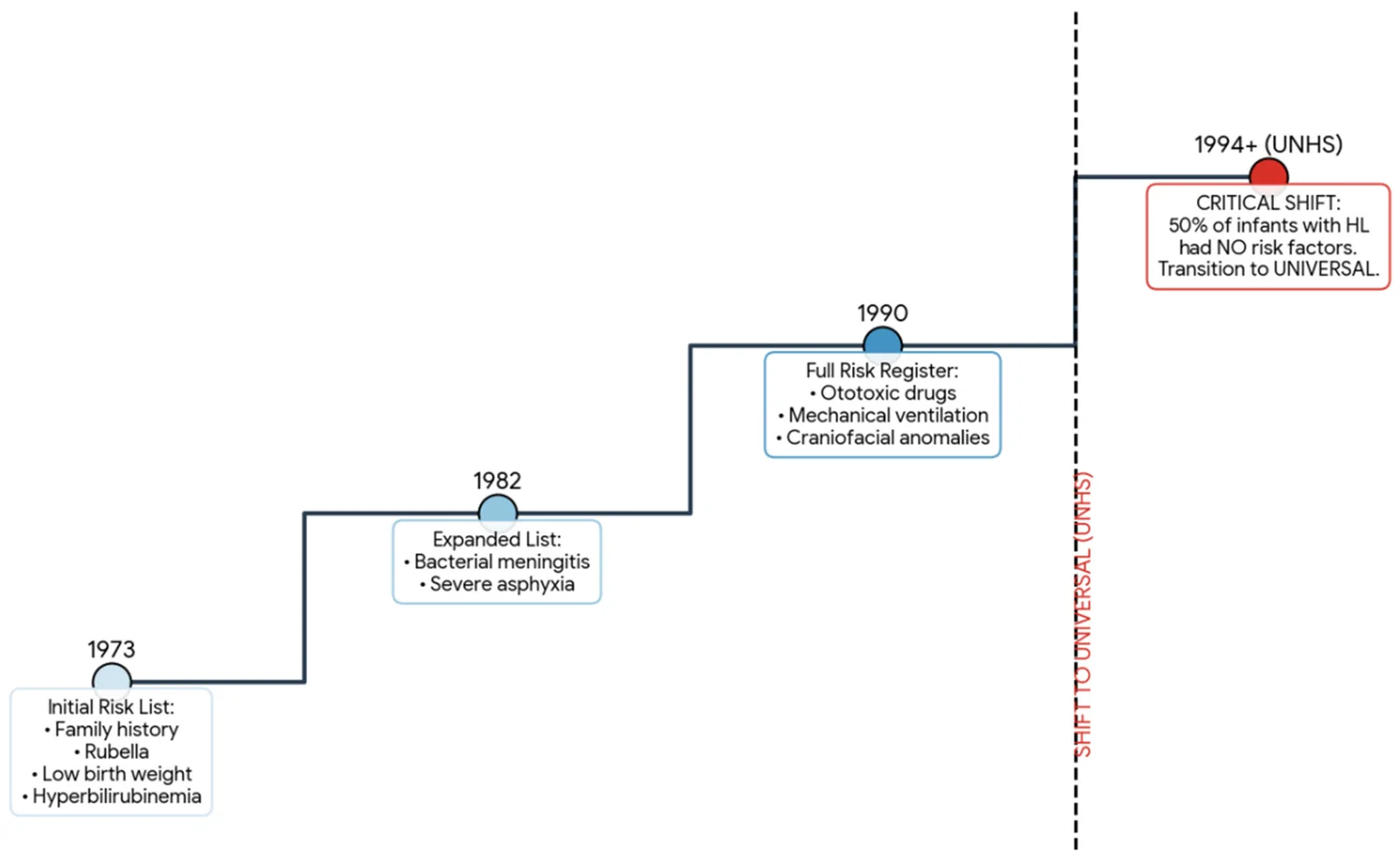
Evolution of newborn hearing screening recommendations and expansion of JCIH risk criteria.

Recognizing the critical importance of hearing, efforts to develop screening programs began as early as 1963 [36]. In 1978, otoacoustic emissions (OAE) testing was introduced for this purpose [37], followed shortly thereafter by auditory brainstem response (ABR), primarily automated ABR (AABR), over the next two years [38]. Initially, screening was limited to newborns with known risk factors for hearing loss. In the 1990s, when it was found that approximately 50% of children with hearing loss were missed despite screening children with known risk factors [39], a universal newborn hearing screening (UNHS) was introduced [12]. After 1994, when the JCIH recommended UNHS for all newborns (Figure 1), diagnoses in countries that followed the recommendation were made more than a year earlier (13.2 months) than in countries without hearing screening [40].

**Figure 2:**
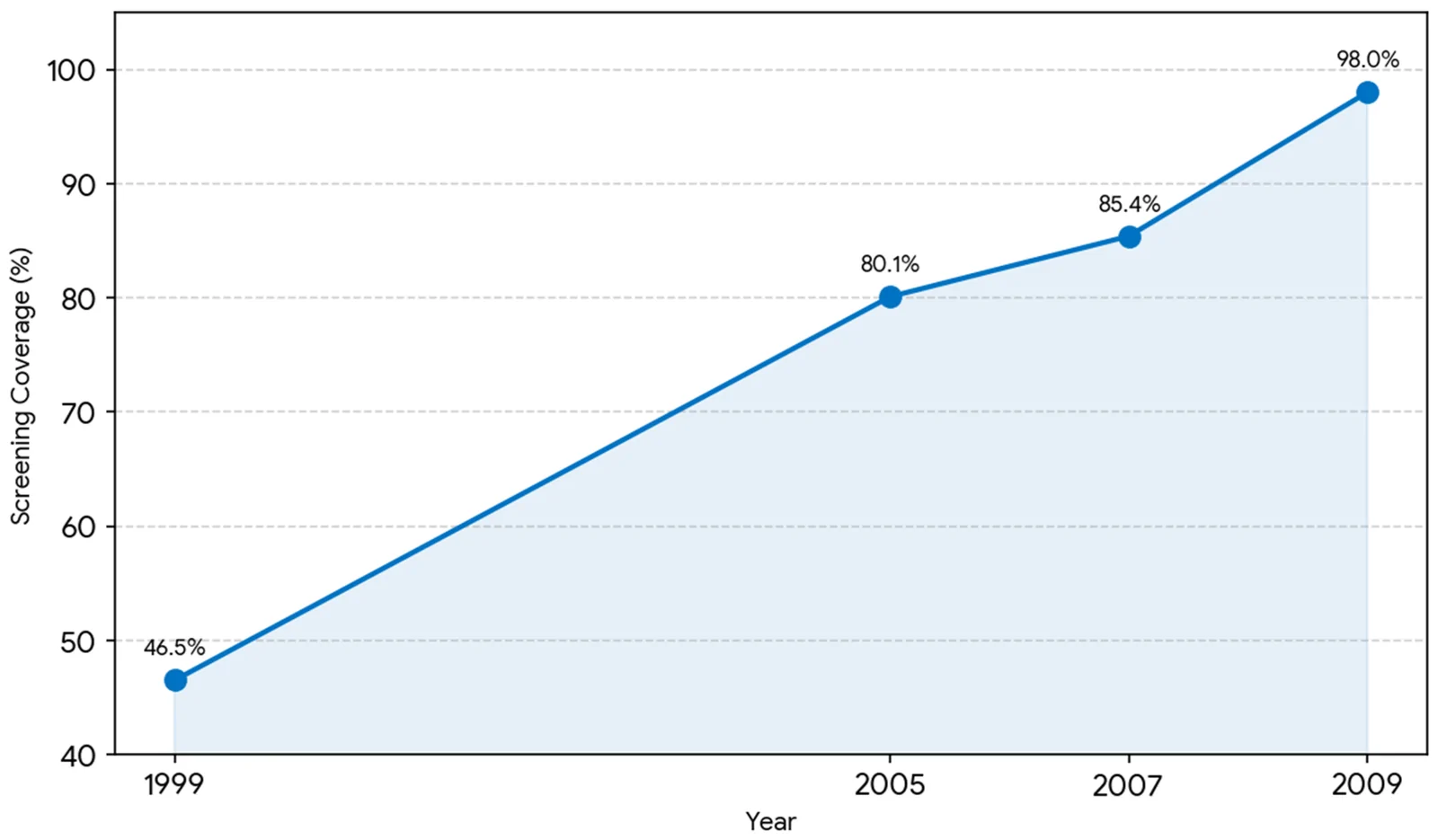
Expansion of universal newborn hearing screening (UNHS) coverage in developed countries (1999–2009). Changes in newborn hearing screening coverage during the first decade of widespread implementation of UNHS. Note: Data adapted from the Early Hearing Detection and Intervention historical reports (1999–2009) and JCIH position statements [41,42].

The universal screening method has spread rapidly in developed countries, as shown in Figure 2. In less developed countries, the program has not yet been properly implemented.

Modern standards for universal hearing screening were established between 2000 and 2010 through the implementation of the Early Hearing Detection and Intervention (EHDI) system [43]. The 1-3-6 rule was established: screening by 1 month, diagnosis by 3 months, and intervention by 6 months.

**Figure 3:**
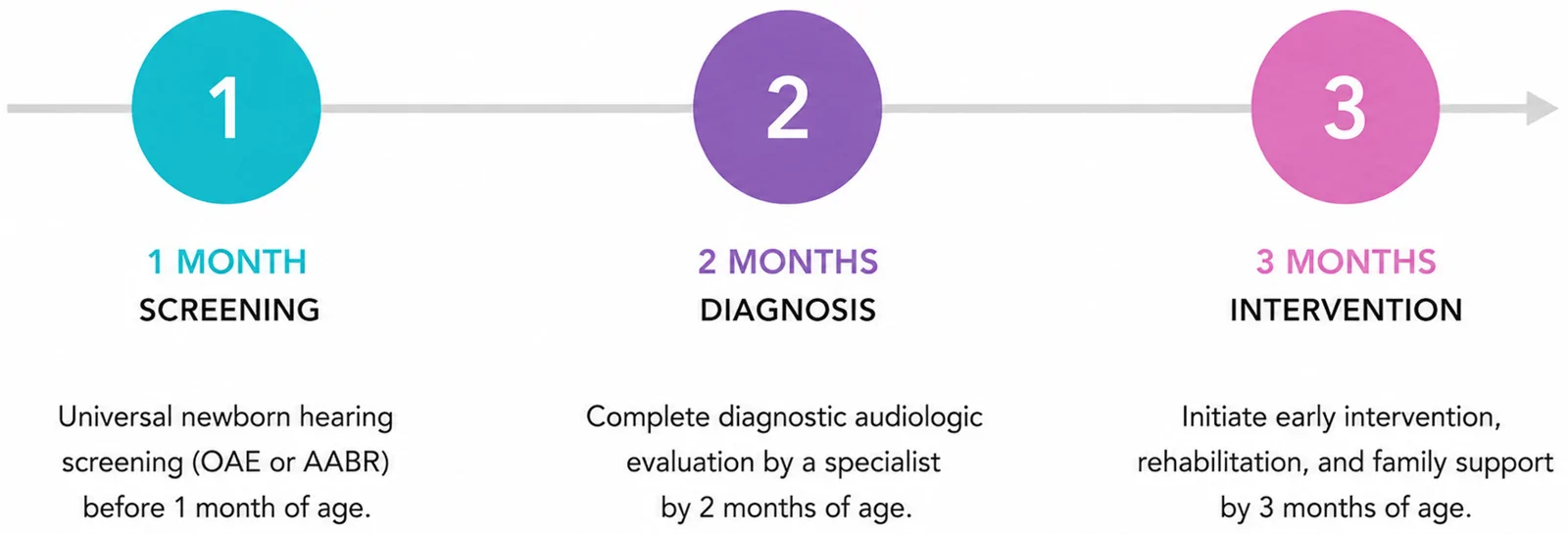
Updated early hearing detection pathway based on the 1–2–3 principle.

According to the 2023 JCIH guidelines, newborn assessment timelines are now even shorter, as shown in Figure 3. The guidelines recommend this timeframe for high-efficiency systems, which enable earlier stimulation of the child’s neurolinguistic development [44,45].

The main reason for earlier intervention is to optimize neuroplasticity in children’s brains. Recent studies have shown that children who receive hearing aids or begin treatment as early as 3 months of age, rather than 6 months, achieve significantly better outcomes in vocabulary development, pronunciation, and later literacy, because this minimizes the period of auditory deprivation for the brain [46].

The progressive implementation of UNHS has substantially improved the early detection of congenital hearing loss worldwide. Nevertheless, despite continuous refinement of screening protocols and recommendations, several diagnostic limitations remain unresolved, particularly regarding the identification of hidden and delayed-onset hearing loss. These limitations are discussed in the following section.

## 5. Diagnostic Limitations of Newborn Hearing Screening

Although universal newborn hearing screening has significantly improved the early identification of congenital hearing loss, no current screening protocol can detect all forms of hearing impairment. Diagnostic failures may result in either false-positive or false-negative outcomes, each associated with different underlying mechanisms and clinical consequences. False-positive results primarily arise from transient conductive conditions or physiological immaturity, whereas false-negative results are more often associated with neural, genetic, infectious, or progressive forms of hearing loss. The following sections summarize the principal mechanisms responsible for these diagnostic limitations.

### 5.1. Mechanisms Responsible for False-Positive Screening Results

- Fluid accumulation in the outer and middle ear

Fluid accumulation in the outer ear (amniotic fluid or vernix) and middle ear (secretory otitis media) can temporarily cause conductive hearing loss [47,48]. These conditions are particularly common in the early postnatal period and among newborns hospitalized in NICU due to medical problems [49]. In healthy newborns, the most common cause is amniotic fluid in the ear canal; in NICU infants, however, due to prolonged hospitalization and an increased risk of middle ear infection, effusive otitis media is also a contributing factor [50]. The likelihood of middle ear effusion in newborns increases with the duration of NICU care and rises exponentially after six months, reaching 13–15% by one year of age [51]. Middle ear effusion at birth is a harmless condition that can be completely resolved; however, prolonged effusion can lead to chronic inflammation and permanent hearing loss [52]. When fluid is present in the middle or outer ear, the recorded hearing threshold can worsen by 40–50 dB due to poorer sound transmission to and from the inner ear [53].

Fluid in the ear also interferes with the objectivity and accuracy of AABR interpretation. After the tested ear is stimulated with a click, AABR records the response along the auditory pathway from the inner ear to the cerebral cortex [54]. There may be several causes of this interference, some of which are similar to those for OAE: stimulus attenuation. Fluid traveling to the inner ear acts as a filter that reduces the intensity of the stimulus (click) reaching the cochlea. This shifts wave latency on the AABR recording in the same way as an inner-ear or auditory-nerve defect, making a differential diagnosis impossible without additional tests [55].

Masking of sensorineural hearing loss by conductive hearing loss [55] can occur. The sensorineural defect remains undetected because the AABR test does not differentiate between conductive and sensorineural hearing loss; it only indicates the overall degree of hearing loss and does not specify whether the hearing loss is solely conductive or combined [56]. It does not allow determination of a bone-conduction hearing threshold curve, which would reveal this impairment. Only an ABR performed in an audiology clinic can do so if the specialist suspects hidden sensorineural hearing loss due to actual damage to the auditory nerve or cochlea [57]. If the examiner mistakenly attributes an abnormal AABR test solely to transient fluid in the outer or middle ear, this may delay the detection of potential permanent sensorineural hearing loss [58]. Tympanometry, which can also be performed in a newborn if middle ear effusion is suspected, is the diagnostic method most likely to mislead the examiner into considering only conductive hearing loss as the cause of the abnormal AABR response [59].

- Anatomical Factors

Anatomical peculiarities of the outer ear—such as the small size of the ear canal and the specific morphology of the outer ear, particularly in preterm infants—affect the objectivity of OAE. Therefore, tympanometry, in addition to other measurements, should be mandatory for all newborns, as it can indicate whether the newborn’s ear canal volume is sufficient for the OAE result to be considered objective.

- Transient dysfunction of hair cells in inner ear

Temporary dysfunction of sensory cells in the inner ear causes transient hearing loss and abnormal screening results, which later resolve. This transient dysfunction of the outer auditory system is due to oxidative stress associated with perinatal complications and asphyxia [60]. Hypoperfusion and cellular hypoxia occur, leading to metabolic changes in the peripheral auditory sensor. An oxidative insult causes transient dysfunction of the outer hair cells. This change is usually reversible, and the hearing threshold typically normalizes within 6 months. Outer hair cells are sensitive to acute oxidative stress but are capable of regeneration [61]. However, because the screening result shows a diminished or absent response of the auditory system to an auditory stimulus on OAE testing, this finding is often interpreted as a sign of permanent sensorineural hearing loss.

Inner hair cells can also regenerate after oxidative stress; however, they are exposed to a different type of stress—not the acute trauma experienced by external sensory organs, but chronic stress. In newborns, the source of chronic stress is intrauterine moderate hypoxia arising from more than one cause. The stress response in inner hair cells often occurs at the synapses. Even before the cell itself dies, stress can disrupt the connections between inner hair cell and the auditory nerve—a condition known as synaptopathy. This is where inner hair cells differ from outer hair cells, in which stress more rapidly leads to direct structural damage and apoptosis (programmed cell death). The repair mechanisms are well understood [61].

- Auditory pathway immaturity

AABR provides a more comprehensive assessment of the auditory pathway than OAE and enables detection of neural disorders, including ANSD [13]. However, interpretation of results is highly dependent on the degree of nervous system maturity, particularly within the auditory pathway. Auditory pathway myelination significantly influences wave morphology, especially V-wave latency and interlatency times [29]. In preterm infants, insufficient myelination or chronic intrauterine hypoxia can lead to apparently pathological findings [8].

Similarly, hyperbilirubinemia can transiently affect AABR results. In all three conditions, there is a high probability of spontaneous recovery [62], and they are only rarely irreversible [63].

In high-risk newborns, therefore, reliable interpretation often requires longer-term follow-up, which may last several months and, in special cases, even until the child reaches 18 months of age [62].

### 5.2. Mechanisms Responsible for False-Negative Screening Results

Although false-positive results add to the burden on the healthcare system, false-negative results pose a greater challenge. Newborns with normal screening results may later develop clinically significant hearing loss [64].

A recent study confirms that a significant number of children develop delayed-onset or progressive hearing loss only in early childhood. Because these children had normal hearing test results at birth, the clinical consequences for speech and academic performance are often quite severe before anyone even suspects a hearing problem [65].

- The underlying mechanisms for such cases can be:
- Mild hearing loss

It has been shown that 23% of infants at 9 months of age have mild hearing loss, despite having undergone a two-stage well-baby hearing screening with normal results [66].

- Genetic disorders

Genetic factors account for approximately 60% of all congenital hearing impairments, with autosomal recessive non-syndromic forms being the most common [18]. Mutations in genes such as *GJB2* and *SLC26A4* exhibit variable expression and incomplete penetrance and can also cause late-onset hearing loss that is not sufficiently pronounced at birth to be detected by screening devices. The impairment only becomes apparent during the school years [67]. Fluctuations in hearing and progression of the impairment are caused by mutations in the *SLC26A4* gene, which disrupt the ionic balance in the inner ear, leading to fluctuating and progressive hearing loss that may be triggered by only a minor head injury or physical exertion during childhood [68,69].

The limitations of current diagnostic strategies pose an additional clinical challenge, as newborn screening tests cannot detect all children with mutations in the *GJB2* gene family. Research shows that the frequency of incomplete penetrance (absence of clinical signs at birth) in children with a *GJB2* gene defect is approximately 3.8% [70]. In practice, this means that 3.8% of newborns who carry this genetic defect will pass universal screening with a normal result, as their auditory pathways are still functional in the first days of life. However, because the connexin 26 protein functions improperly, ionic homeostasis in the inner ear is eventually disrupted, leading to gradual, progressive hearing loss. Such impairment manifests clinically and is diagnosed only later in childhood, when it reaches a stage that noticeably affects the child’s communication, thereby confirming the necessity of long-term audiological monitoring of these children [22].

- Congenital cytomegalovirus infection

Congenital cytomegalovirus infection is the most common non-genetic cause of progressive and late-onset hearing loss [71], as well as the most common cause of non-genetic hearing loss in children, accounting for 21% of detectable hearing loss in newborns and 25% of hearing loss in four-year-olds. The overall prevalence of cCMV infection among newborns is 0.64% [72]. cCMV infection may be symptomatic (11%) or asymptomatic (90%) at birth [72]. The symptomatic form carries a significantly higher risk of hearing loss, with a 40% to 50% probability, while the asymptomatic form has a 10% to 15% probability [73]. Although the symptomatic form carries a poorer prognosis for the child’s hearing, we immediately perform diagnostic procedures to evaluate hearing loss, given the high prevalence of hearing impairment. In the asymptomatic form, however, the infection is often not even recognized, let alone the possibility of hearing loss, and therefore the diagnosis of hearing loss can be significantly delayed. Once symptomatic cCMV infection is confirmed within the first month after birth, it can be treated very successfully over the next six months with the antiviral drug ganciclovir. The congenital form of the infection is very difficult to detect after the third week of life [74].

Hearing loss associated with cCMV infection varies widely, from mild to severe impairment. It can be progressive, it can occur with a delay, and, most challenging for accurate diagnosis, it can also be fluctuating. Therefore, data on the prevalence of cCMV-associated hearing loss are highly controversial. The progressive form is thought to occur in 7% to 70% of asymptomatic congenital infections [75]. The delayed-onset form can develop up to age 5, and the fluctuating form up to age 16. As many as 40% of cCMV-related hearing impairments are therefore overlooked [76]. Even if cCMV affects hearing in only one ear, the likelihood of subsequent progressive pathology in the other ear is as high as 12.5% [77].

- Auditory neuropathy spectrum disorder

ANSD is a significant cause of hidden hearing loss. It is characterized by preserved outer hair cell function (hence normal otoacoustic emissions) and disturbed signal transmission along the auditory pathway (abnormal or absent ABR response) [78].

This is a defect in the connection between the detection and perception of an auditory signal, where standard screening tests for hearing thresholds identify the detection defect but not the perception defect.

Defects can occur at several levels:

- At the level of the inner ear: the conversion of the sound’s mechanical energy into a nerve signal is impaired, resulting in a weaker signal traveling along the auditory nerve;
- At the synapse between the inner ear and the auditory nerve (auditory synaptopathy). The synapse has a highly complex structure, as it contains highly specialized ribbon vesicles on the side facing the inner ear. These vesicles can synchronously release a neurotransmitter that encodes the sound signal in both time and amplitude, enabling precise transmission of auditory information to the auditory cortex. Because of this complex structure, numerous genetic defects are possible on both sides of the synapse;
- At the level of the auditory nerve [79].

**Figure 4:**
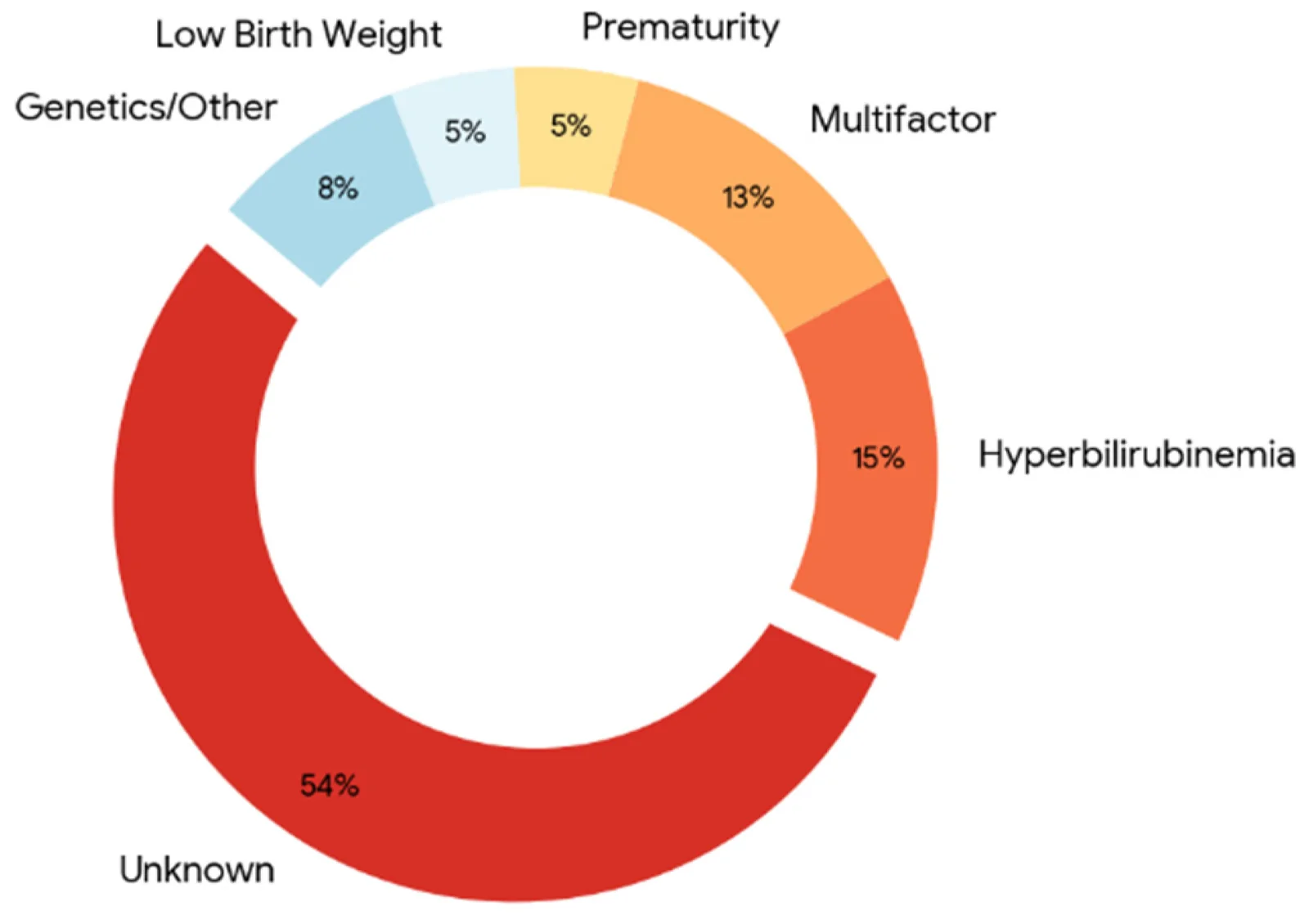
Estimated distribution of the principal causes of auditory neuropathy spectrum disorder.

In more than half of cases, the etiology of ANSD is unknown (see Figure 4).

Estimates of the prevalence of ANSD vary across pediatric populations. In the general pediatric population, the prevalence is 1 per 10,000 [80], while among children hospitalized in the NICU, it is 0.2%. Among children diagnosed with sensorineural hearing loss, 6.5% have ANSD [81].

## 6. Approaches to Reduce the Number of Misinterpreted Screening Results

Limitations of hearing screening (fluid buildup in the outer or middle ear, anatomical factors, and transient sensorineural hearing loss in the inner ear) are reflected in the proportion of children who require further diagnostic evaluation—the so-called referral rate. The referral rate indicates the proportion of newborns with an abnormal screening test result who are referred for further audiological evaluation. With OAE screening, the referral rate is relatively high (22–50%), especially if the OAE is performed within the first 24–72 h after birth. The first factor mentioned, amniotic fluid in the ear canal, is responsible for the abnormal result [82]. As the time from birth to time of screening increases, the referral rate decreases significantly, to approximately 4–10% after the third day of life [83,84]. It remains constant until around the 30th day, then rises again because of the possibility of fluid in the middle ear. A high referral rate is very disruptive for parents, as it causes unnecessary worry, and for the healthcare system, as it results in unnecessary examinations and costs. Therefore, a rule has been established that the referral rate should be less than 4%, intended to reflect the minimal presence of problematic circumstances [82].

Crucial for reducing the referral rate due to misinterpreted screening results are appropriate timing of screening and two-stage screening protocols. Long-term monitoring is essential for a definitive assessment of hearing status in children with suspected hearing loss.

### 6.1. Appropriate Timing of Screening

To achieve the desired referral rate of 4%, which is considered to represent true indications and would not be the result of transient conductive hearing loss, screening is recommended between 2 and 20 days of age for well -babies and between 5 and 13 days of age for NICU infants. Studies have shown that the referral rate reaches a realistic level by the 3rd day after birth [85].

The timing of the test is also essential because the degree of auditory nerve myelination and synaptogenesis varies among newborns [86]. Interpretation of AABR results is challenging due to immature myelination, which can lead to transient abnormal findings [35]. Incorrect timing can result in an AABR result in a newborn with an immature auditory pathway being identical to that of a newborn with ANSD, even though this result will be normal in a few months once maturation is complete. During the morphogenesis of the auditory pathway, or any part of the nervous system, maturation processes must be coordinated with one another. If they are not, arrest or delay occurs. It is difficult to predict which process is in the arrest phase [87].

Poor timing of the maturation processes can lead to neurological dysfunction. This can be corrected once the processes are synchronized, a phenomenon known as reversibility. One-third of newborns in intensive care are affected by delayed maturation, which is reversible on average after four months [88].

This category also includes reversible hearing loss, which was observed in cases of hearing loss of less than 70 dB in as many as 69.4% of patients after four to six months of observation. This finding is also concerning for cochlear implant therapy [89,90].

### 6.2. The Use of Two-Stage Screening Protocols

Possible combinations of tests include OAE and OAE, OAE and AABR, AABR and OAE, and AABR and AABR [91]. Meta-analyses of two-stage screening tests have demonstrated their effectiveness, particularly in head-to-head comparisons of the following combinations: OAE-OAE, OAE-AABR, AABR-OAE, and AABR-AABR. Overall, two-stage screening has drastically reduced the referral rate, although the extent of this reduction depends on the specific test combination. For example, protocols in both stages of testing that are performed in the same manner (AABR-AABR or OAE-OAE) are more effective (referral rate 1.3% and 2.7%, respectively) and have a lower referral rate than those using a mixed combination such as AABR-TEOAE (5.9%) [91]. Table 1 shows the clinical implications of the various test combinations.

Nevertheless, the OAE test remains the most commonly performed—and often the first stage of—newborn screening because it is simple, safe, and rapid. It is affordable, easy to interpret, and serves as a sensitive indicator of hearing loss. Furthermore, it does not require highly specialized personnel. It is particularly easy to perform in this age group, as newborns are less restless than older infants, eliminating the need for sedation [64].

The AABR test is performed as a second-stage test only if the OAE result is abnormal. AABR is not routinely performed in healthy newborns [64,92,93]. Two-stage protocols (OAE–AABR) are well established for healthy newborns, whereas the AABR–AABR combination is more commonly used for newborns in the NICU because of the higher prevalence of ANSD [94]. AABR remains the first-stage screening test for children with risk factors and for children hospitalized in the intensive care unit [58]. In these cases, the test is performed before discharge.

Compared with OAE, AABR yields fewer false-positive results, which is important in the population of children from the NICU, as they are at higher risk for neurogenic hearing loss due to a variety of risk factors that are present even before admission to the NICU or that develop during their stay in the NICU [19,94].

### 6.3. Follow-Up Care for Children with Suspected Hearing Impairment

Although two-stage testing has been shown to reduce referral rates, it has important limitations. The OAE-OAE protocol does not detect ASND, and the AABR-AABR protocol is time-consuming and costly. Experts have noted that while two-stage tests help filter out transient conductive artifacts, they also substantially increase the risk of false-negative results and the likelihood that parents will not bring their child for follow-up visits. The core problem is securing children’s return for follow-up after hearing impairment has been suspected [19].

Because the combination of OAE and AABR is most commonly used in two-stage screening, errors in detecting hearing loss occur most often with this protocol. Olusanya confirms that although the combination of OAE and AABR is the gold standard for detecting typical congenital hearing loss, both technologies systematically miss mild, late-onset, and hidden hearing loss (cochlear synaptopathy), as they measure only pure-tone thresholds within a narrow frequency range [95].

A second longitudinal study documents cases of children who passed initial OAE and ABR tests in the maternity ward but later developed sensorineural hearing loss or showed clear signs of it. The authors emphasize that a “normal” screening result does not rule out functional hearing difficulties in noisy environments [64].

OAE measures the function of the outer hair cells in the cochlea, while AABR measures the presence of a neural wave at a specific sound level. Neither test assesses synaptic density or the quality of transmission of complex acoustic signals. Screening is conducted in a completely quiet environment. However, hidden hearing loss manifests clinically only in noisy settings (daycare centers, schools), when the impaired synaptic pathway cannot cope with ambient noise [96].

Several studies have shown that a significant proportion of NICU infants with pathological AABR findings later exhibit substantial improvement or even normalization of auditory responses [89]. Delayed auditory maturation appears particularly common in premature infants and neonates exposed to hyperbilirubinemia or perinatal asphyxia [97]. In some cohorts, reversible auditory abnormalities have been observed in nearly one-third of high-risk infants, with significant improvement occurring within the first four to six months of life [89].

Similar reversible neural dysfunction has been reported in infants with hypoxic–ischemic injury and perinatal asphyxia [96]. These findings strongly support the view that early abnormal AABR findings do not necessarily indicate irreversible neural pathology [97].

Long-term follow-up is essential for all children with any prior suspicion of hearing loss, whether clinical or based on test findings, because screening test outcomes can change over time.

## 7. Proposal to Expand Screening and Recommendations for Clinical Practice

The future of successful detection and management of children with hearing loss will require upgrading existing protocols through continuous, systematic monitoring of children’s functional hearing throughout the preschool and school years, as well as greater access to audiological rehabilitation, which remains a key public health goal worldwide [31].

**Figure 5:**
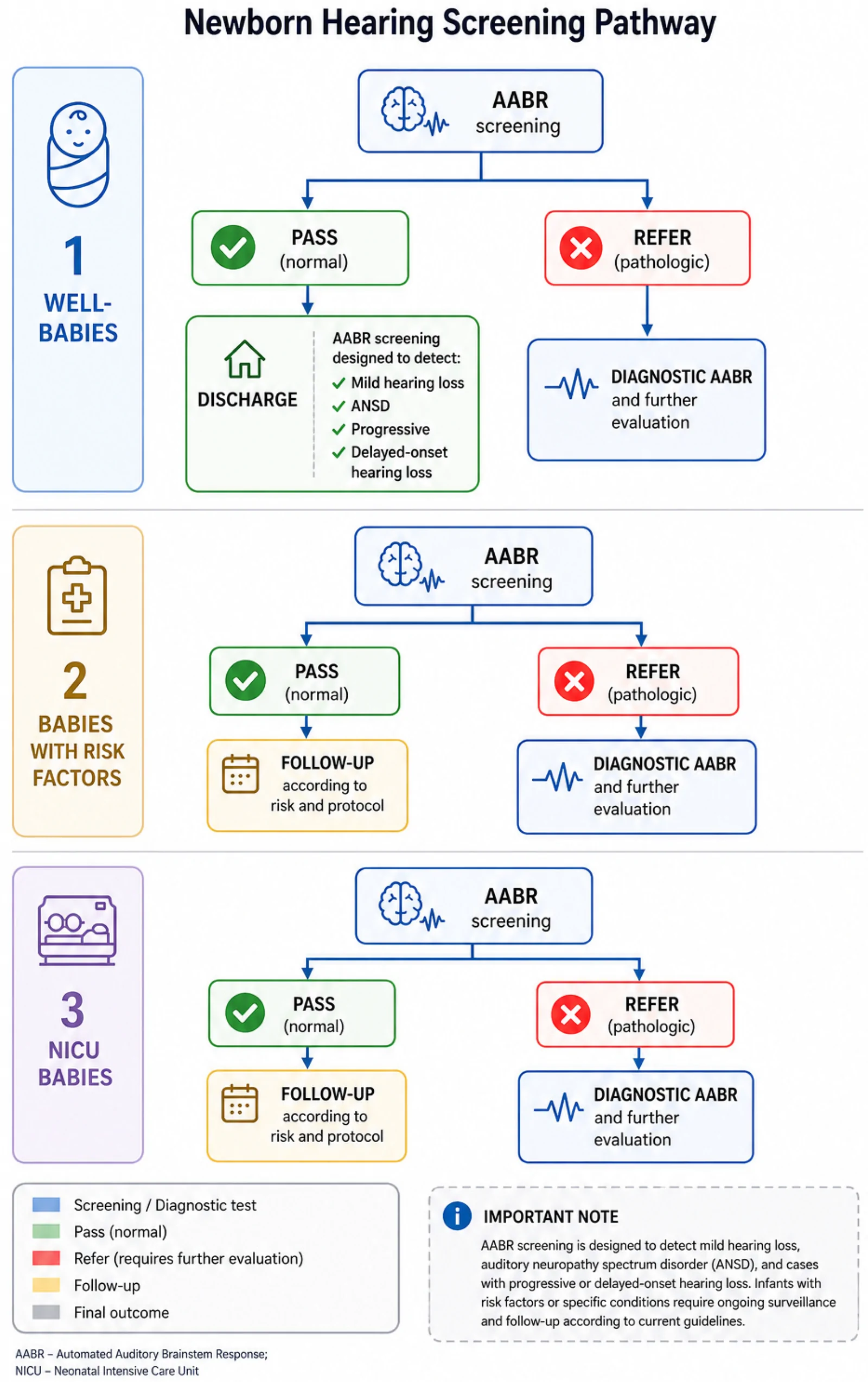
Proposed comprehensive newborn hearing screening algorithm. Infants with risk factors require continued audiological surveillance regardless of initial screening outcome. Abbreviations: AABR, automated auditory brainstem response; ANSD, auditory neuropathy spectrum disorder; NICU, neonatal intensive care unit.

Table 2 and Figure 5 present our recommendations for newborn hearing screening.

Based on data from the literature, we can summarize the following clinical recommendations for including an assessment of the entire auditory system in screening programs:

- Replacing standard OAE screening with mandatory AABR and tympanometry;
- CMV testing at birth for all live-born infants;
- *GJB2* and *SLC26A4* gene testing at birth for all live-born infants;
- Minimizing the impact of technical and physical factors on false-positive and false-negative screening results (examination timing, use of tympanometry, two-stage screening);
- Active screening for mild and unilateral hearing loss among otherwise normal children, as well as progressive, fluctuating, and delayed-onset genetic and infectious hearing impairments;
- Follow-up monitoring of the child’s speech development in natural environments (in noise).

## 8. Future Perspectives

### 8.1. Current Knowledge Gaps

Despite the remarkable success of universal newborn hearing screening (UNHS), several important gaps remain in the current evidence. First, there is still no universally accepted screening protocol capable of reliably identifying all forms of hidden hearing loss, including auditory neuropathy spectrum disorder (ANSD), mild hearing loss, progressive genetic hearing loss, delayed-onset hearing loss, and congenital cytomegalovirus (cCMV)-associated hearing loss. Existing screening strategies rely predominantly on physiological testing and often fail to identify disorders that become clinically apparent only months or years after birth.

Second, the natural history of many delayed-onset hearing disorders remains poorly understood. Longitudinal studies investigating the progression of hearing impairment in children with genetic mutations, cCMV infection, or early electrophysiological abnormalities are still limited. Consequently, the optimal duration and frequency of audiological follow-up remain uncertain.

Another important knowledge gap concerns auditory pathway maturation in newborns. Delayed myelination may mimic pathological ABR findings and contribute to both false-positive and false-negative screening outcomes. More reliable biomarkers capable of distinguishing physiological immaturity from permanent neural dysfunction are therefore urgently needed.

Finally, there is relatively little evidence regarding the cost-effectiveness of integrating genetic testing, cCMV screening, and advanced electrophysiological assessment into routine newborn hearing screening programs. There are major unresolved issues in the current literature, including the limited detection of hidden and delayed-onset hearing loss, uncertainties regarding long-term follow-up strategies, the challenges associated with auditory pathway maturation, and the lack of evidence regarding the integration of molecular diagnostics into routine screening programs.

### 8.2. Research Priorities

Future research should focus on developing more comprehensive and individualized hearing screening strategies. Large prospective multicenter studies are needed to evaluate whether universal first-line AABR screening improves the detection of hidden hearing loss compared with current OAE-based protocols.

Particular attention should be directed toward combining physiological screening with molecular diagnostics. Studies evaluating targeted or universal genetic screening, especially for genes such as *OTOF*, *GJB2*, and *SLC26A4*, together with early cCMV testing, may substantially improve the identification of infants at risk for delayed-onset hearing loss.

Another important research priority is the development of predictive risk models that integrate clinical risk factors, electrophysiological findings, genetic information, and neurodevelopmental indicators. Artificial intelligence and machine learning algorithms may facilitate individualized risk prediction and optimize follow-up strategies.

Future investigations should also establish standardized international follow-up protocols that extend beyond the neonatal period, particularly for infants with recognized risk factors despite normal newborn screening results.

Finally, studies should evaluate long-term developmental outcomes, including speech perception, language acquisition, educational achievement, psychosocial development, and quality of life, rather than focusing exclusively on hearing thresholds.

### 8.3. Potential Impact on Clinical Practice and Public Health

Future advances in newborn hearing screening have the potential to fundamentally transform pediatric audiology. Earlier identification of hidden and delayed-onset hearing loss would enable earlier intervention during the critical period of auditory neuroplasticity, thereby improving speech, language, cognitive, and social development.

From a clinical perspective, integrating AABR, genetic testing, cCMV diagnostics, tympanometry, and individualized longitudinal surveillance could significantly reduce diagnostic uncertainty, decrease false-positive and false-negative results, and improve the selection of appropriate rehabilitation strategies, including hearing aids and cochlear implantation.

At the public health level, improved screening strategies could reduce the long-term burden of untreated childhood hearing loss by decreasing educational support needs, improving academic achievement, increasing future employment opportunities, and reducing healthcare and societal costs. More accurate identification of at-risk children would also facilitate equitable access to early intervention services and improve health outcomes across different populations.

Ultimately, newborn hearing screening may evolve from a single physiological test performed shortly after birth into a precision-based, lifelong hearing surveillance model that integrates physiological, molecular, and clinical data to provide individualized care for every child.

## 9. Conclusions

A narrative review of existing findings on the shortcomings of screening shows that the current trend in UNHS focuses on detecting conventional hearing impairments, primarily severe and profound sensorineural hearing loss, rather than the growing range of diverse auditory dysfunctions that significantly contribute to developmental disorders in children. Nevertheless, UNHS remains the cornerstone of progress in diagnosing hearing loss in children.

In the future, we propose that the strategy for early detection of hearing loss focus not only on determining a single hearing threshold but also on assessing the functionality of the entire auditory system.

Combining electrophysiological testing, genetic, and infectious factors with an assessment of nervous system maturity would improve the accuracy of hearing loss diagnosis and enable more individualized therapeutic strategies.

Current screening programs fail to detect all forms of hearing loss in children. Hidden and delayed-onset forms represent a significant clinical problem that affects the development of speech, cognition, and social skills.

We therefore propose that the JCIH take all these conclusions into account at its next meeting, add AABR as the first stage of universal screening—not only for children in intensive care—and, above all, precisely define the timing of AABR measurements.

The future of newborn hearing screening is likely to move beyond the concept of universal neonatal testing toward precision hearing surveillance. Instead of considering hearing screening as a single event performed shortly after birth, future programs should integrate physiological assessment, molecular diagnostics, congenital CMV screening, longitudinal audiological follow-up, and individualized risk stratification. Such an approach has the potential to substantially reduce the number of children with undetected hearing loss and maximize developmental outcomes through earlier diagnosis and timely intervention.

## Figures and Tables

**Table 1:** Clinical implications of diagnostic inaccuracies in two-stage screening protocols.

Screening Protocol (1st Stage → 2nd Stage)	Consequences of False-Positive (2nd Stage)	Consequences of False-Negative (2nd Stage)
OAE → OAE	High referral rates Prolonged parental anxiety Unnecessary follow-up visits	Missed Auditory Neuropathy Delayed identification of neural hearing loss
OAE → AABR	Unnecessary invasive diagnostics Risk of inappropriate clinical interventions	Missed prelingual deafness Undetected progressive loss (genetic/cCMV) Delayed speech and language development
AABR → OAE	Unnecessary extensive diagnostic workup	Missed prelingual deafness
AABR → AABR	Redundant specialized follow-up procedures	Missed prelingual deafness Significant speech and language delays

Abbreviations: OAE, otoacoustic emissions; AABR, automated auditory brainstem response; cCMV, congenital cytomegalovirus.

**Table 2:** Proposed clinical recommendations for optimizing newborn hearing screening and improving detection of hidden and delayed-onset hearing loss.

Clinical Step	Recommendation	Rationale
Screening protocol	Mandatory AABR screening for all newborns.	OAE does not detect auditory neuropathy, which is 20 times more common in the NICU.
Diagnostics	OAE + ABR + tympanometry combined.	Elimination of false-positive results (effusion) and identification of the ANSD paradox.
Time Frame	Strict adherence to the 1-2-3 rule.	Early intervention during the first 3 months of life is crucial for the development of neural pathways and speech.
Genetics	Referral for genetic testing (e.g., *OTOF* gene) when ANSD is suspected.	The high proportion (54%) of cases with unknown etiology of ANSD requires molecular diagnostics.
Follow-up	Monitor speech development regularly, even if hearing is within the normal range.	In cases of ANSD, the hearing threshold does not reflect the actual ability to understand speech.

## Data Availability

No new data were created or analyzed in this study. Data sharing is not applicable to this article.

## Abbreviations

The following abbreviations are used in this manuscript:

AABR	Automatic Auditory brainstem responses
ABR	Auditory brainstem responses
ANSD	Auditory neuropathy spectrum disease
cCMV	Congenital cytomegalovirus
JCIH	Joint Committee on Infant Hearing
NICU	Neonatal intensive care unit
OAE	Otoacoustic emissions
TOAE	Transitory otoacoustic emission
UNHS	Universal newborn hearing screening
